# Diagnostic accuracy of myocardial perfusion imaging in patients evaluated for kidney transplantation: A systematic review and meta-analysis

**DOI:** 10.1007/s12350-021-02621-x

**Published:** 2021-05-04

**Authors:** Jeroen R. Kelderman, Floris E. J. Jolink, Stan Benjamens, Andrea G. Monroy-Gonzalez, Robert A. Pol, Riemer H. J. A. Slart

**Affiliations:** 1 University of Groningen, Medical Imaging Centre, Department of Nuclear Medicine and Molecular Imaging, University Medical Center Groningen, Groningen, The Netherlands; 2 University of Groningen, Division of Transplant Surgery, Department of Surgery, University Medical Center Groningen, Groningen, The Netherlands; 3 Department of Biomedical Photonic Imaging, Faculty of Science and Technology, University of Twente, Enschede, The Netherlands

**Keywords:** End-stage renal disease, Myocardial perfusion imaging, Invasive coronary angiography, Coronary artery disease, Diagnosis, Systematic review, Meta-analysis

## Abstract

**Background:**

Cardiovascular disease is the most common cause of death after kidney transplantation. Coronary artery disease (CAD) assessment is therefore mandatory in patients evaluated for transplantation. We aimed to assess the diagnostic accuracy for CAD of single-photon emission computed tomography (SPECT) compared to the standards invasive coronary angiography (ICA) and coronary computed tomography angiography (CCTA) in patients evaluated for kidney transplantation.

**Methods:**

We performed a systematic literature search in PubMed, EMBASE, Web of Science, OvidSP (Medline), The Cochrane Library and Google Scholar. Studies investigating the diagnostic accuracy of myocardial perfusion imaging (MPI) SPECT in patients evaluated for kidney transplantation were retrieved. After a risk of bias assessment using QUADAS-2, a meta-analysis was conducted.

**Results:**

Out of 1459 records, 13 MPI SPECT studies were included in the meta-analysis with a total of 1245 MPI SPECT scans. There were no studies available with CCTA as reference. Pooled sensitivity of MPI SPECT for CAD was 0.66 (95% CI 0.53 to 0.77), pooled specificity was 0.75 (95% CI 0.63 to 0.84) and the area under the curve (AUC) was 0.76. Positive likelihood ratio was 2.50 (95% CI 1.78 to 3.51) and negative likelihood ratio was 0.41 (95% CI 0.28 to 0.61). Pooled positive predictive value was 64.9% and pooled negative predictive value was 74.1%. Significant heterogeneity existed across the included studies.

**Conclusions:**

MPI SPECT had a moderate diagnostic accuracy in patients evaluated for kidney transplantation, with a high rate of false-negative findings. The use of an anatomical gold standard against a functional imaging test in the included studies is however suboptimal.

**Supplementary Information:**

The online version contains supplementary material available at 10.1007/s12350-021-02621-x.

## Introduction

Mortality after kidney transplantation has been importantly reduced in the last three decades, particularly in the early post-transplantation period. However, mortality by cardiovascular disease remains an important risk in the first three months after surgery.^1^ This is not unexpected, as reports of invasive coronary angiography (ICA) in patients with end-stage renal disease (ESRD) evaluated for kidney transplantation document a prevalence of coronary artery disease (CAD) between 42% and 81%.^2^

In ESRD patients evaluated for kidney transplantation, there is a poor correlation between clinical presentation and significant CAD as only 44% of dialysis-dependent patients with acute myocardial infarction present with chest pain, compared to 68% of patients in the general population.^3^ As such, the presentation of CAD is frequently asymptomatic, making it difficult to identify patients that may benefit from medical therapies to reduce the CAD burden.^3^,^4^ These patients may benefit from cardiovascular screening techniques for asymptomatic CAD by reducing the procedural risk during a kidney transplantation.

The current gold-standard method for detecting CAD is ICA; however, the invasive nature of this technique makes it only appropriate to perform in the presence of high likelihood of obstructive CAD. An alternative technique is coronary computed tomography angiography (CCTA), a non-invasive method used in patients with low clinical likelihood of obstructive CAD.^2^,^5^ Unfortunately, both methods can induce contrast nephropathy in ESRD patients,^2^,^6^ and CT is often of limited value in ESRD patients due to the high atherosclerotic burden.^2^

In patients with intermediate likelihood of obstructive CAD, there is considerable experience with non-invasive myocardial perfusion imaging (MPI), using single-photon emission computed tomography (SPECT). For the general population, MPI SPECT is considered a reliable diagnostic technique for the functional detection of CAD, as it provides a qualitative and semi-quantitative assessment of the myocardial perfusion defect.^7^,^8^

We aimed to assess the diagnostic accuracy of SPECT for CAD assessment compared to ICA and CCTA, in patients evaluated for kidney transplantation.

## Methods

This systematic review and meta-analysis was performed according to the Preferred Reporting Items for Systematic Reviews and Meta-Analyses of Diagnostic Test Accuracy Studies (PRISMA-DTA) statement.^9^ The PRISMA 2009 checklist table is available as a supplement of this article (S1 Appendix). The study protocol is registered with PROSPERO, protocol number CRD42020188610.

### Literature Search

A literature search was performed by two reviewers (JK, FJ) using the following databases: PubMed, EMBASE, Web of Science, OvidSP (Medline), The Cochrane Library and Google Scholar. Detailed search strategies, including Medical Subject Headings (MeSH)-terms and Emtree terms, are available as a supplement of this article (S2 Appendix). A medical information specialist examined and verified our search strategies for all the databases. All identified records published until 5th March 2021 were exported.

### Selection of Records

Obtained records were entered into the evidence synthesis tool CADIMA.^10^ After removal of duplicates by CADIMA, the records were independently screened on title and abstract. Randomized controlled trials and observational studies in patients evaluated for kidney transplantation were included if they compared MPI SPECT to ICA or CCTA. Papers written in a non-English language, papers where the full text was not available, non-human trials, studies with a small sample size (*n* < 30), conference abstracts, editorials, protocol papers and systematic reviews were excluded. Any inconsistencies were resolved after discussion with an independent third reviewer (SB).

### Quality Assessment

QUADAS-2, a tool developed for the systematic review of diagnostic accuracy studies, was used to assess risk of bias and applicability of a study.^11^ The criteria of the QUADAS-2 tool were: bias due to selection, index test, reference test, and flow and timing. The index tests were MPI and the reference standards were ICA or CCTA. The quality assessment was performed independently by the two reviewers (JK and FJ). Disagreements were resolved by discussing with a third reviewer (SB).

All studies were rated as having a high, low or intermediate/ uncertain risk of bias. Uncertain risk of bias was graded in the following cases: for the domain ‘risk of bias due to patient selection’ if the studies performed SPECT MPI and ICA only in patients at high risk for CAD, for the domains ‘risk of bias due to index test’ or ‘risk of bias due to reference standard’ if it was unsure whether blinding was performed, and for the domain ‘risk of bias due to flow and timing’ if it was unclear how much time there was between the index test and reference test, and if not all patients underwent both tests.

### Data Extraction

Using a structured template, study characteristics (i.e., design and sample size), baseline population demographics (i.e., age, sex, diabetes mellitus), used definitions for abnormal cardiac testing, and the reported outcomes of individual studies were extracted.

### Statistical Methods

For the diagnostic studies, sensitivity and specificity forest plots were created in RevMan, Version 5.4 (The Cochrane Collaboration, 2020, Copenhagen, Denmark). A bivariate analysis and receiver operating characteristic (ROC) curve were created for the summary estimate and the corresponding 95% CI with R software package: A Language and Environment for Statistical Computing, version 1.0.153 for Mac (R Foundation for Statistical Computing, Vienna, Austria). Heterogeneity was visually checked in forest plots and the summary receiver operating curve (SROC). An *I*^2^ higher than 50% was considered indicative of significant study heterogeneity.^12^ Negative and positive likelihood ratios, as well as diagnostic odds ratios (DOR), were calculated with Open Meta-Analyst (OpenMeta [Analyst], Brown School of Public Health, Providence, USA).

## Results

### Studies Included

**Fig. 1:**
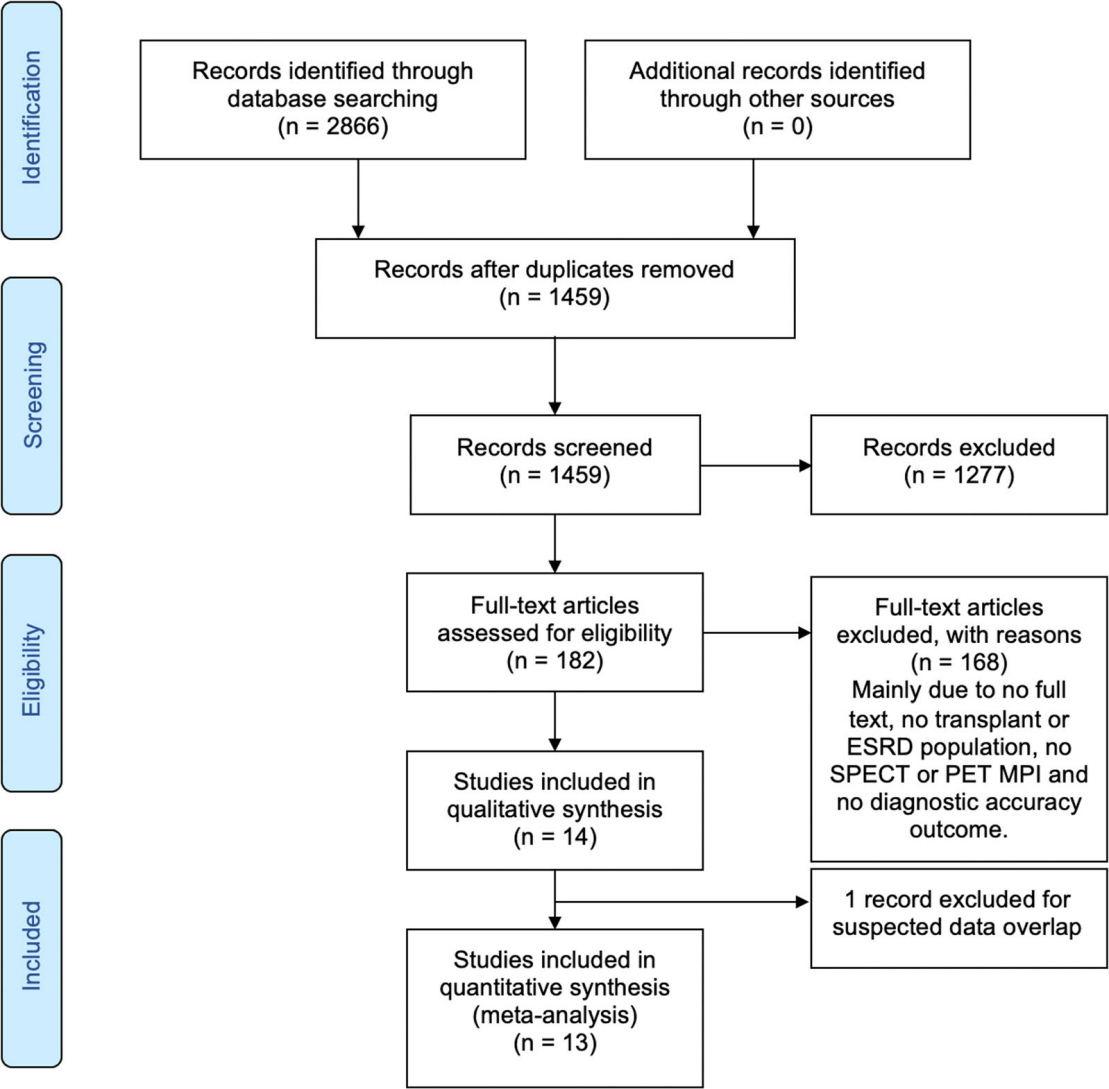
Flowchart of included studies

After duplicate removal, we identified 1459 studies, published between 1990 and 2019. After screening, 13 studies were eligible for qualitative analysis, all targeting MPI SPECT. No studies could be included using the reference standard CCTA (Fig. 1). Ten studies were prospective and three were retrospective. We excluded one study after full-text screening because of suspected data overlap with the study of Winther et al.^13^

### Study Characteristics

A total of 1245 MPI SPECT scans and 1258 ICAs were performed. MPI SPECT images were labeled as abnormal based on the presence of fixed or reversible perfusion defects or the calculated summed stress score. ICA results were labeled as abnormal and a CAD diagnosis was made based on the percentage of stenosis. Five studies used > 50% stenosis as the cut-off, six studies used > 70% stenosis or more as the cut-off and two studies used a combination of > 50% and > 70% stenosis to determine CAD. The study by Wilson et al.^14^ used > 50% for the left main coronary artery, > 70% for epicardial coronary arteries and 50% to 70% in borderline lesions. The study by Doukky et al.^15^ used > 50% for the left main coronary artery and >70% stenosis in any of the epicardial coronary arteries (Table 1).

**Table 1:** Characteristics of studies included in the meta-analysis

Authors	Year	Country	Study design	No. of MPI scans	No. of ICA or CCTA scans	Mean age (years)	% male	Definition of abnormal MPI	Definition of abnormal stenosis on ICA or CCTA	Radiotracer type
Atkinson et al.^26^	2011	UK	P	47	47	n/a	79.0%	Reversible or fixed defect	> 50% stenosis	Technetium-99m tetrofosmin
Boudreau et al.^27^	1990	USA	P	80	80	38	64.0%	Reversible or fixed defect	> 70% stenosis	Thallium-201
De Lima et al.^28^	2003	Brazil	P	122	106	55	77.0%	Reversible or fixed defect	> 70% stenosis	Technetium-99m-sestamibi
De Lima et al.^29^*	2010	Brazil	P	228	228	56	71.0%	Reversible or fixed defect	> 70% stenosis	Technetium-99m-sestamibi
Doukky et al.^15^	2018	USA	R	89	90	55	61.0%	SSS > 4	> 50% and > 70% stenosis	Technetium-99m tetrofosmin
Enkiri et al.^30^	2010	USA	P	57	57	54	53.0%	Reversible or fixed defect	> 50% stenosis	Technetium-99m-sestamibi
Garg et al.^31^	2000	India	P	19	52	46	88.5%	Reversible or fixed defect	> 50% stenosis	Thallium-201
Marwick et al.^32^	1990	USA	P	45	45	49	62.0%	Reversible or fixed defect	> 50% stenosis	Thallium-201
Vandenberg et al.^33^	1996	USA	R	41	47	37	n/a	Reversible or fixed defect	> 75% stenosis	Thallium-201
Welsh et al.^34^**	2011	Canada	P	245	234	48	67.1%	Reversible or fixed defect	> 70% stenosis	Technetium-99m-sestamibi
Wilson et al.^14^	2019	USA	R	94	94	53	60.0%	Reversible or fixed defect	> 50% and > 70% stenosis	Thallium-201, Technetium-99m-tetrofosmin
Winther et al.^13^	2015	USA	P	138	138	54	68.1%	SSS > 3	> 50% stenosis	Technetium-99m-sestamibi
Worthley et al.^35^	2003	Australia	P	40	40	50	48.0%	Reversible or fixed defect	> 70% stenosis	Technetium-99m-tetrofosmin

*CCTA*, coronary computed tomography angiography, *ICA*, invasive coronary angiography, *MPI*, myocardial perfusion imaging, *P*, prospective, *R*, retrospective, *SSS*, summed stress score

*The study by de Lima et al.^29^ presents two cohorts, sufficient data were only available for the validation cohort

**The study by Welsh et al.^34^ used > 50% and > 70% as definition for significant CAD. However, diagnostic accuracy was only calculated for > 70% stenosis

### Quality ASSESSMENT

The results of the QUADAS-2 tool are summarized in Table 2.

**Table 2:** QUADAS-2 risk of bias assessment

Authors	Year	Bias domain				Applicability		
		Patient selection	Index test	Reference standard	Flow and timing	Patient selection	Index test	Reference standard
Atkinson et al.^26^	2011	Low	Low	Low	Uncertain	Low	Low	Low
Boudreau et al.^27^	1990	Uncertain	Low	Low	Uncertain	Low	Low	Low
De Lima et al.^28^	2003	Uncertain	Uncertain	Low	Uncertain	Low	Low	Low
De Lima et al.^29^	2010	Uncertain	Low	Uncertain	Uncertain	Low	Low	Low
Doukky et al.^15^	2018	Uncertain	Low	High	High	Low	Low	Low
Enkiri et al.^30^	2010	Uncertain	Low	Low	Uncertain	Low	Low	Low
Garg et al.^31^	2000	Uncertain	Low	Low	Uncertain	Low	Low	Low
Marwick et al.^32^	1990	Uncertain	Low	Low	Low	Low	Uncertain	Uncertain
Vandenberg et al.^33^	1996	Uncertain	Low	Low	Low	Low	Uncertain	Uncertain
Welsh et al.^34^	2011	Uncertain	Uncertain	Uncertain	Uncertain	Low	Low	Low
Wilson et al.^14^	2019	Low	Low	Low	Low	Low	Low	Low
Winther et al.^13^	2015	Low	Low	Low	Low	Low	Low	Low
Worthley et al.^35^	2003	Uncertain	Low	Low	Uncertain	Low	Low	Low

### Technical Aspect

Three different radiopharmaceuticals were used in the studies: Technetium-99m-sestamibi, Technetium-99m-tetrofosmin and Thallium-201. All studies performed imaging on conventional Anger gamma camera systems. MPI SPECT/CT imaging was not used, the included studies used MPI SPECT only. The software programs used in the included studies were not mentioned.

### Diagnostic Accuracy

**Fig. 2:**
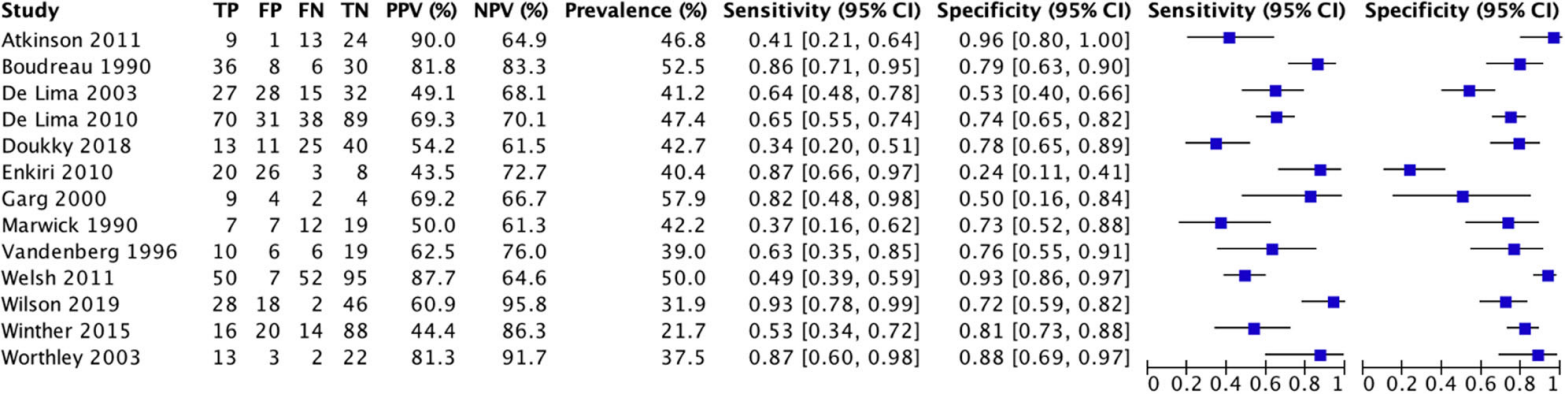
Sensitivity and specificity of myocardial perfusion imaging SPECT for coronary artery disease in patients evaluated for kidney transplantation

Diagnostic data were extracted from all included studies (*n* = 13). Three studies performed MPI SPECT as part of standard care, ten studies only in high-risk patients. Sensitivity of the included studies ranged from 34% to 93% and specificities ranged from 24% to 96% (Fig. 2).

**Fig. 3:**
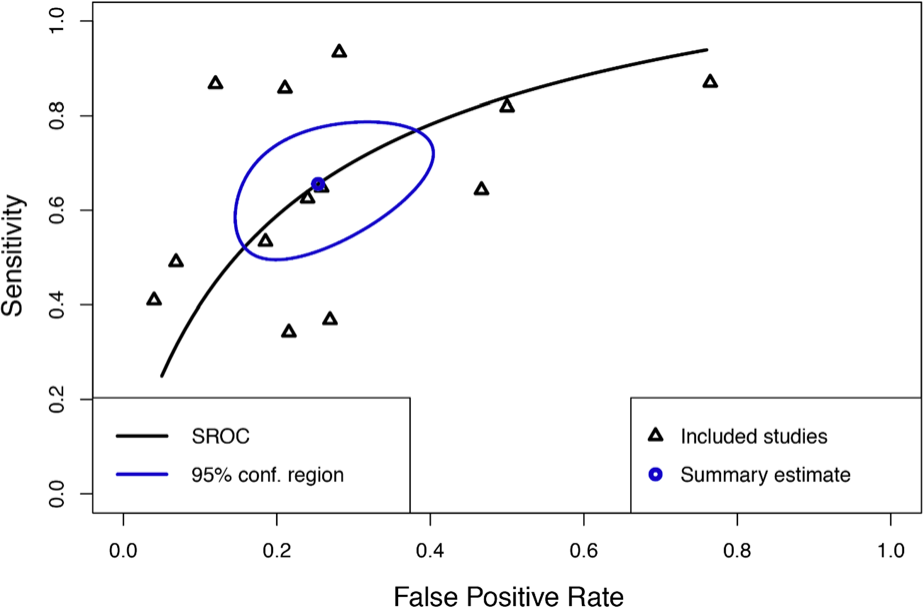
Summary Receiver Operating Curve (SROC) of included studies

**Fig. 4:**
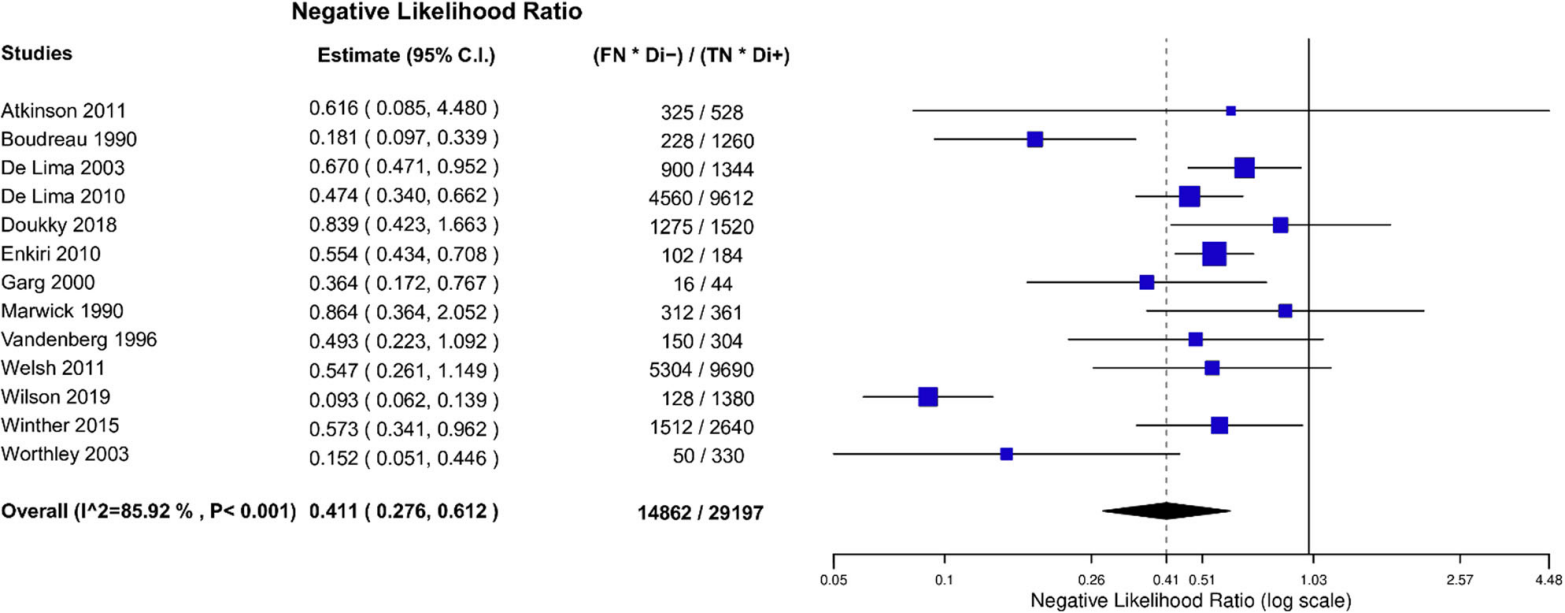
Negative Likelihood Ratio (NLR) of included studies

**Fig. 5:**
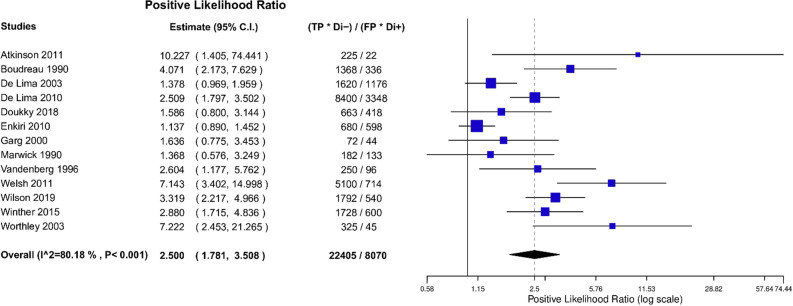
Positive Likelihood Ratio (PLR) of included studies

**Fig. 6:**
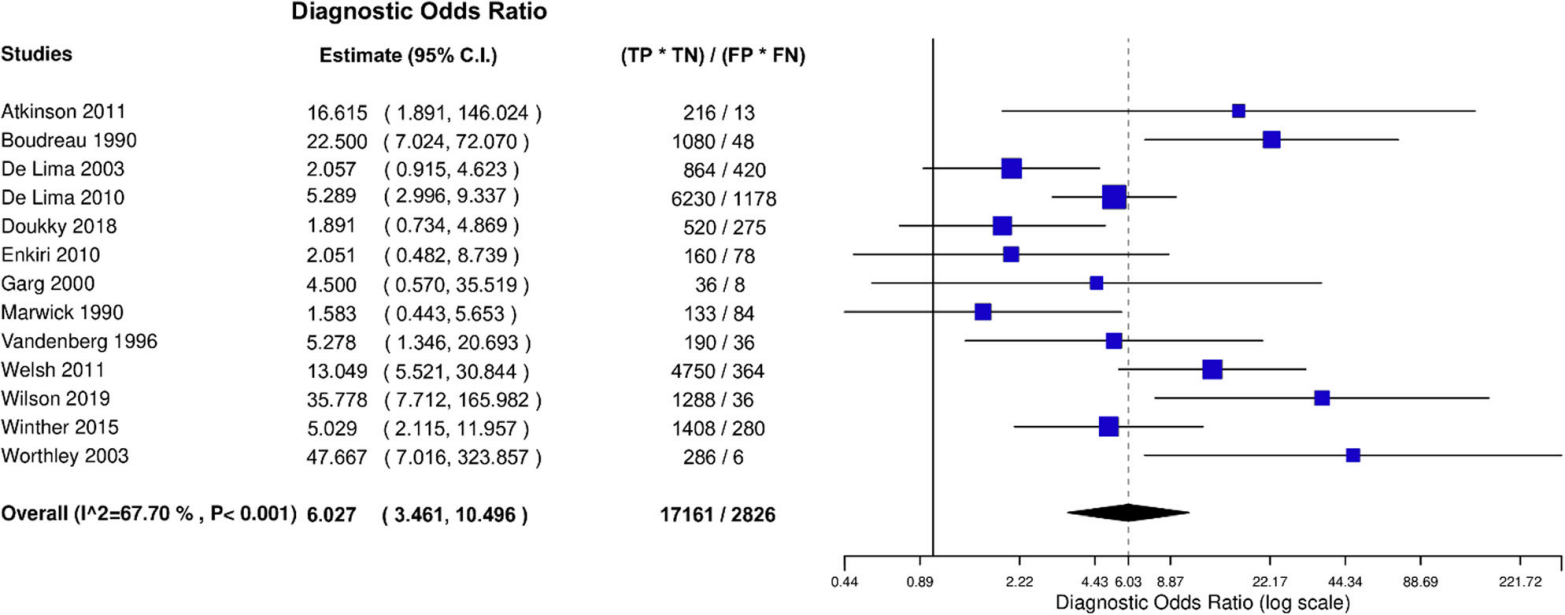
Diagnostic Odds Ratio (DOR) of included studies

After bivariate analysis a mean sensitivity for MPI SPECT of 0.66 (95% CI 0.53 to 0.77), mean specificity of 0.75 (95% CI 0.63 to 0.84) and an area under the curve (AUC) of 0.76 (Fig. 3) was calculated. Positive likelihood ratio (PLR) was 2.50 (95% CI 1.78 to 3.51) and negative likelihood ratio (NLR) was 0.41 (95% CI 0.28 to 0.61) with an overall diagnostic odds ratio (DOR) of 6.03 (95% CI 3.46 to 10.50) (Figs. 4, 5, 6). Pooled positive predictive value was 64.9%, pooled negative predictive value was 74.1%, and pooled CAD prevalence was 42.4%. There was evidence of heterogeneity present in the forest plots for sensitivity and specificity (Figs. 2, 4, 3). We found significant heterogeneity (*I*^2^ > 50%) for PLR, NLR, and DOR (Figs. 4, 5, 6).

## Discussion

This systematic review and meta-analysis evaluated the ability of MPI SPECT to detect CAD in patients evaluated for kidney transplantation. With data from 13 studies and a combined total of 1245 MPI SPECT scans, we demonstrated that MPI SPECT had only moderate sensitivity and specificity, ranging between 34% and 93% and 24% to 96%, respectively, for detecting clinically relevant CAD. A high heterogeneity existed across the included studies and there were no studies available with CCTA as reference.

Although the available studies do not show a high accuracy of MPI SPECT for the diagnose of CAD, current guidelines recommend screening with MPI SPECT in patients with cardiovascular risk factors.^16^,^17^ A joined statement by the international cardiology and transplantation societies on cardiac disease evaluation in kidney and liver transplantation candidates, recommends non-invasive stress testing when three or more CAD risk factors are present (diabetes mellitus, prior cardiovascular disease, duration of dialysis of > 1 year, left ventricular hypertrophy, age > 60 years, smoking, hypertension and dyslipidemia), regardless of functional status (Class IIb, Level of Evidence C).^17^

A meta-analysis on the diagnostic accuracy of MPI SPECT for the detection of CAD in the general population reported a pooled sensitivity and specificity of, respectively, 0.88 (95% CI 0.88 to 89) and 0.61 (95% CI 0.59 to 0.62).^18^ A previous systematic review by Wang et al., determined the diagnostic accuracy of MPI SPECT in ESRD patients. Their study showed a pooled sensitivity of 0.74 (95% CI 0.54 to 0.87) and specificity of 0.70 (95% CI 0.51 to 0.84),^19^ which is comparable to the outcomes presented in the current study. When comparing MPI SPECT in patients with ESRD to the general population, a lower sensitivity but high specificity is observed. Patients with ESRD often have hypertension, left ventricular hypertrophy and decreased coronary flow reserve, all of which may account for reduced sensitivity of MPI SPECT in kidney transplant candidates, and may explain the differences with the general population.^20^ In patients with end-stage liver disease (ESLD), a meta-analysis compared the diagnostic accuracy of MPI SPECT with adenosine and regadenoson as vasodilating agents. This study reported a pooled sensitivity of 0.62 (95% CI 0.44 to 0.79) and pooled specificity of 0.82 (95% CI 0.77 to 0.87) for detecting severe CAD with adenosine scans. For regadenoson, these numbers were, respectively, 0.35 (95% CI 0.14 to 0.62) and 0.88 (0.82 to 0.92).^21^ The sensitivity of MPI SPECT in ESLD patients may be lower than in the general population due to hemodynamic changes and reduced coronary vascular resistance.^22^ Therefore, there might be an impaired response to agents inducing pharmacological stress. The pooled sensitivity and specificity of MPI SPECT for CAD reported in the current study (0.66 and 0.75, respectively) is comparable to the results in patients with ESRD and ESLD, with a lower sensitivity and higher specificity compared to MPI in the general population. Several studies demonstrated the association of perfusion defects on MPI SPECT and cardiovascular events following kidney transplantation. In an analysis of 1189 renal transplant patients, of which 819 underwent MPI SPECT, Ives et al. reported that abnormal MPI SPECT findings is an independent risk factor of cardiovascular events after renal transplantation (Hazard Ratio 1.78 (95% CI (1.03 to 3.06)).^23^ In a retrospective cohort study with 401 patients who underwent MPI SPECT prior to kidney transplantation, Doukky et al. reported that MPI SPECT had long-term prognostic value in patients with 3 or more AHA/ACCF risk factors.^15^ Therefore, MPI SPECT can be used as well as a screening tool to predict cardiovascular events in patients evaluated for renal transplant.

The current review demonstrated that all of the 13 included studies applied visual/semi-quantitative scoring, which resulted in significant heterogeneity across the studies. In the included studies, there was a lack of a standard SPECT procedure, such as a wide variety of radiopharmaceuticals, one- or two-day stress protocols, different types of specialists who assess the imaging findings, differences in pharmacological stress inducers, different software programs, and variation in time interval between injection and MPI SPECT. This may well explain the wide confidence intervals found for sensitivities, specificities, PLR and NLR. This underlines that a standardization of the protocols is warranted which will improve the quality and reproducibility of MPI SPECT. Thus currently the procedural guidelines for cardiac MPI SPECT issued by the European Association of Nuclear Medicine (EANM) are recommended.^24^

MPI PET has several advantages compared to MPI SPECT. Due to the routine correction of radiotracer attenuation, the higher spatial resolution, the higher extraction of PET perfusion tracers, PET scanning has a better diagnostic accuracy than SPECT.^25^ The lower radiation burden and the ability to make an absolute quantitative perfusion assessment with PET is another advantage of this method.^25^ However, PET scanning is relative more expensive and less widely available. Production of the short-lived radiopharmaceuticals requires a costly cyclotron or a generator. The diagnostic accuracy of PET has to date not been investigated in the ESRD population although a higher accuracy is expected for the functional diagnosis of CAD when using MPI PET in these patients.

Our study has some limitations that need to be addressed, including those inherent to systematic reviews and meta-analyses of diagnostic test accuracy studies. We included studies before the year 2000 and as with technology, radioisotopes and MPI SPECT procedures have changed and improved over time. This may have influenced the results of the included studies.

MPI SPECT is used to assess the functional significance of CAD and for risk stratification before renal transplantation. On the contrary, the reference standard ICA uses anatomy to assess CAD. An equivalent comparison between MPI SPECT and ICA has its limitations. Selecting 0.89% of the published studies on the subject since 1990 may have potential bias of including highly selected centers. Strengths of this work are the extensive literature search in the six most recognized databases, reporting according to the PRISMA-DTA statement,^9^ study protocol registration with PROSPERO and a structured and validated bias assessment using the QUADAS-2 tool.^11^

## Conclusion

This systematic review and meta-analysis demonstrated that MPI SPECT had a moderate diagnostic accuracy in patients evaluated for kidney transplantation and resulted in a high rate of false-negative findings. The use of an anatomical gold standard against a functional imaging test is however suboptimal. Further research is essential to establish the role of standardized MPI SPECT for the evaluation of patients prior to transplantation, with special attention for the new dynamic MPI SPECT implementation or replacement by MPI PET.

## New Knowledge Gained

Application of MPI SPECT to detect CAD in patients evaluated for kidney transplantation has been performed. According to our study results MPI SPECT had a moderate diagnostic accuracy for functional CAD in patients evaluated for kidney transplantation as compared to a suboptimal anatomical standard and offers room for technical imaging improvements.

## Supplementary Information

Below is the link to the electronic supplementary material.

Electronic supplementary material 1 (DOCX 28 kb)

Electronic supplementary material 2 (DOCX 27 kb)

Electronic supplementary material 3 (PPTX 918 kb)

## Abbreviations

ICA: Invasive coronary angiography
ESRD: End-stage renal disease
ESLD: End-stage liver disease
CCTA: Coronary computed tomography angiography
SPECT: Single-photon emission computed tomography
MPI: Myocardial perfusion imaging
PET: Positron emission tomography
CACS: Coronary artery calcium score
PLR: Positive likelihood ratio
NLR: Negative likelihood ratio
DOR: Diagnostic odds ratio
AUC: Area under the curve
