# Supplementary material for: Diagnostic accuracy of myocardial perfusion imaging in patients evaluated for kidney transplantation: A systematic review and meta-analysis
Source: J Nucl Cardiol. 2021 May 4;29(6):3405–15. doi: 10.1007/s12350-021-02621-x (PMC9834355; doi:10.1007/s12350-021-02621-x)
Supplement: Supplementary file 2 — Electronic supplementary material 2 (DOCX 27 kb) [file 12350_2021_2621_MOESM2_ESM.docx]

**Appendix 2**

**PubMed**

| (  "kidney transplantation"[mesh] OR  "kidney transplant*"[tiab] OR  "renal transplant*"[tiab] OR  "end stage renal disease*"[tiab] OR  (  (  “kidney failure, chronic”[mesh] OR  "chronic kidney disease*"[tiab]  ) AND  "transplant*"[tiab]  )  ) AND  (  "myocardial perfusion imaging"[mesh] OR  "mpi"[tiab] OR  "perfusion imaging"[tiab] OR  "myocardial perfusion"[tiab] OR  "coronary artery disease/diagnostic imaging"[mesh] OR  "myocardial scintigraph*"[tiab] OR  "thallium" [mesh] OR  "thallium" [tiab] OR  (  (  "heart"[tiab] OR  "cardiac"[tiab] OR  "cardiovasc*"[tiab] OR  "myocard*"[tiab] OR  "coronary"[tiab]  ) AND  (  "tomography, emission-computed"[mesh] OR  "spect"[tiab] OR  "tomograph*"[tiab] OR  "pet"[tiab]  )  )  ) |
| --- |

**Embase**

| **#1:**  (  "kidney transplantation"/exp OR  "kidney transplant*":ab,ti OR  "renal transplant*":ab,ti OR  "end stage renal disease*":ab,ti OR  (  (  "chronic kidney disease*":ab,ti OR  "chronic kidney failure"/exp  ) AND  "transplant*":ab,ti  )  ) AND  (  "myocardial perfusion imaging"/exp OR  "mpi":ab,ti OR  "perfusion imaging":ab,ti OR  "myocardial perfusion":ab,ti OR  "myocardial scintigraph*":ab,ti OR  "thallium"/exp OR  "thallium":ab,ti OR  (  (  "heart":ab,ti OR  "cardiac":ab,ti OR  "cardiovasc*":ab,ti OR  "myocard*":ab,ti OR  "coronary":ab,ti OR  "coronary artery disease"/exp  ) AND  (  "computer assisted emission tomography"/exp OR  "spect":ab,ti OR  "tomograph*":ab,ti OR  "pet":ab,ti  )  )  )  **#2:** #1 AND 'conference abstract'/it  **#3:** #1 NOT #2 |
| --- |

**Web of Science**

| TS=(  "kidney transplant*" OR  "renal transplant*" OR  "end stage renal disease*" OR  (  (  "chronic kidney disease*" OR  "chronic kidney failure"  ) AND  "transplant*"  )  ) AND  TS=(  "myocardial perfusion imaging" OR  "mpi" OR  "perfusion imaging" OR  "myocardial perfusion" OR  "myocardial scintigraph*" OR  "thallium" OR  (  (  "heart" OR  "cardiac" OR  "cardiovasc*" OR  "myocard*" OR  "coronary"  ) AND  (  "spect" OR  "tomograph*" OR  "pet"  )  )  ) |
| --- |

**OvidSP (Medline)**

| (  "kidney transplant*" OR  "renal transplant*" OR  "end stage renal disease*" OR  (  (  "chronic kidney disease*" OR  "chronic kidney failure"  ) AND  "transplant*"  )  ) AND  (  "myocardial perfusion imaging" OR  "mpi" OR  "perfusion imaging" OR  "myocardial perfusion" OR  "myocardial scintigraph*" OR  "thallium" OR  (  (  "heart" OR  "cardiac" OR  "cardiovasc*" OR  "myocard*" OR  "coronary"  ) AND  (  "spect" OR  "tomograph*" OR  "pet"  )  )  ) |
| --- |

**The Cochrane Library**

| **#1:** [mh "kidney transplantation"]  **#2:** "kidney transplant*":ti,ab,kw OR "renal transplant*":ti,ab,kw OR "end stage renal disease*":ti,ab,kw OR ("chronic kidney disease*":ti,ab,kw AND "transplant*":ti,ab,kw)  **#3:** [mh "myocardial perfusion imaging"]  **#4:** [mh "coronary artery disease"/DG]  **#5:** "mpi":ti,ab,kw OR "perfusion imaging":ti,ab,kw OR "myocardial perfusion":ti,ab,kw OR "myocardial scintigraph*":ti,ab,kw  **#6:** "heart":ti,ab,kw OR "cardiac":ti,ab,kw OR "cardiovasc*":ti,ab,kw OR "myocard*":ti,ab,kw OR "coronary":ti,ab,kw OR "thallium":ti,ab,kw  **#7:** [mh "tomography, emission-computed"]  **#8:** "spect":ti,ab,kw OR "tomograph*":ti,ab,kw OR "pet":ti,ab,kw  **#9:** (#1 OR #2) AND (#3 OR #4 OR #5 OR (#6 AND (#7 OR #8))) |
| --- |

**Google Scholar**

| allintitle: ("Kidney transplantation" OR “kidney transplant” OR “renal transplant” OR “renal transplantation”) AND ("myocardial perfusion" OR "myocardial scintigraphy" OR "perfusion imaging" OR “SPECT” OR “PET”)) |
| --- |
