# Supplementary material for: Diagnostic accuracy of myocardial perfusion imaging in patients evaluated for kidney transplantation: A systematic review and meta-analysis
Source: J Nucl Cardiol. 2021 May 4;29(6):3405–15. doi: 10.1007/s12350-021-02621-x (PMC9834355; doi:10.1007/s12350-021-02621-x)
Supplement: Supplementary file 3 — Electronic supplementary material 3 (PPTX 918 kb) [file 12350_2021_2621_MOESM3_ESM.pptx]

## Slide 1
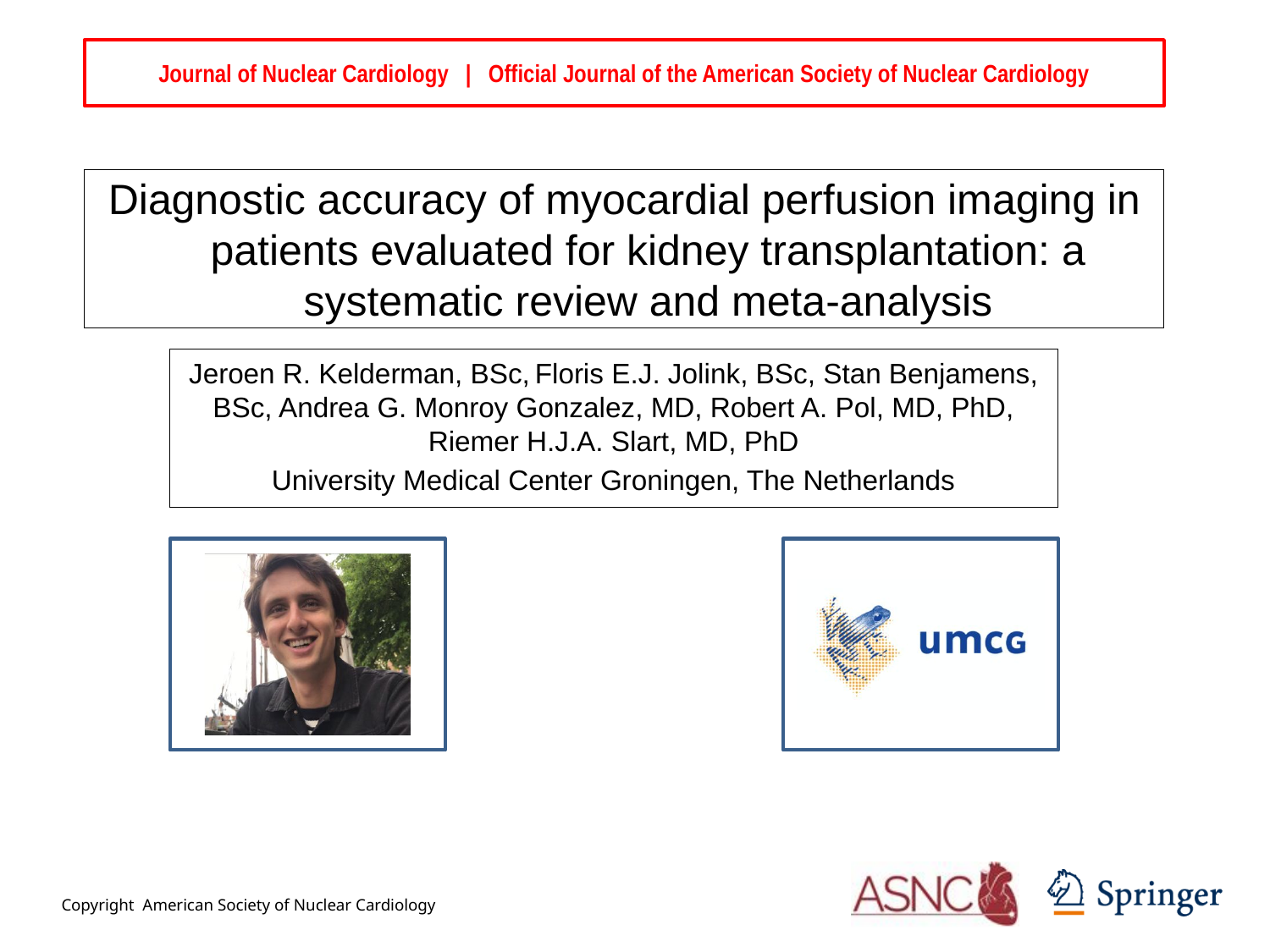

Journal of Nuclear Cardiology | Official Journal of the American Society of Nuclear Cardiology
# Diagnostic accuracy of myocardial perfusion imaging in patients evaluated for kidney transplantation: a systematic review and meta-analysis
Jeroen R. Kelderman, BSc, Floris E.J. Jolink, BSc, Stan Benjamens, BSc, Andrea G. Monroy Gonzalez, MD, Robert A. Pol, MD, PhD, Riemer H.J.A. Slart, MD, PhD
University Medical Center Groningen, The Netherlands
Head shot of author
required
Copyright American Society of Nuclear Cardiology

## Slide 2
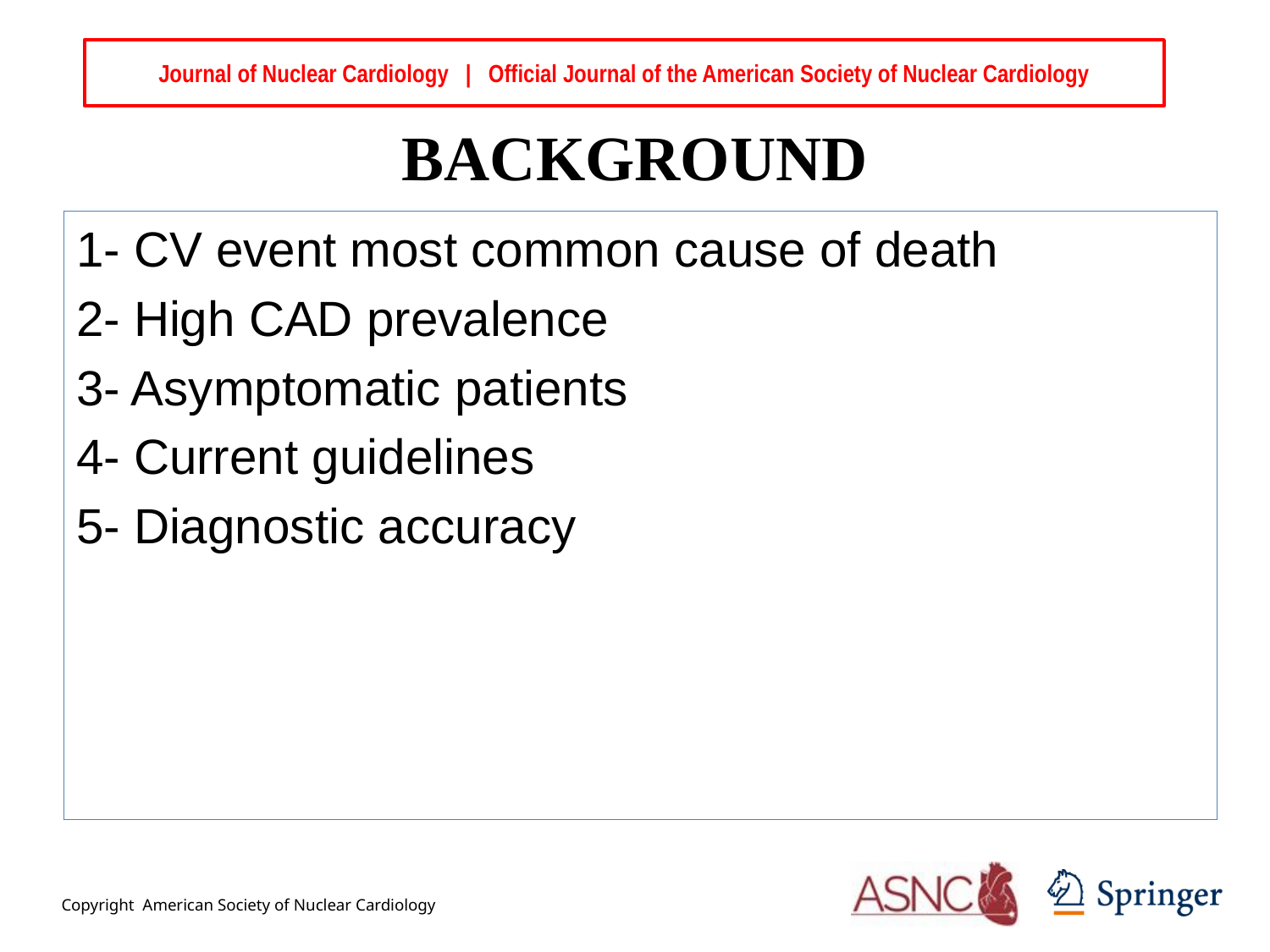

Journal of Nuclear Cardiology | Official Journal of the American Society of Nuclear Cardiology
# BACKGROUND
1- CV event most common cause of death
2- High CAD prevalence
3- Asymptomatic patients
4- Current guidelines
5- Diagnostic accuracy
Copyright American Society of Nuclear Cardiology

## Slide 3
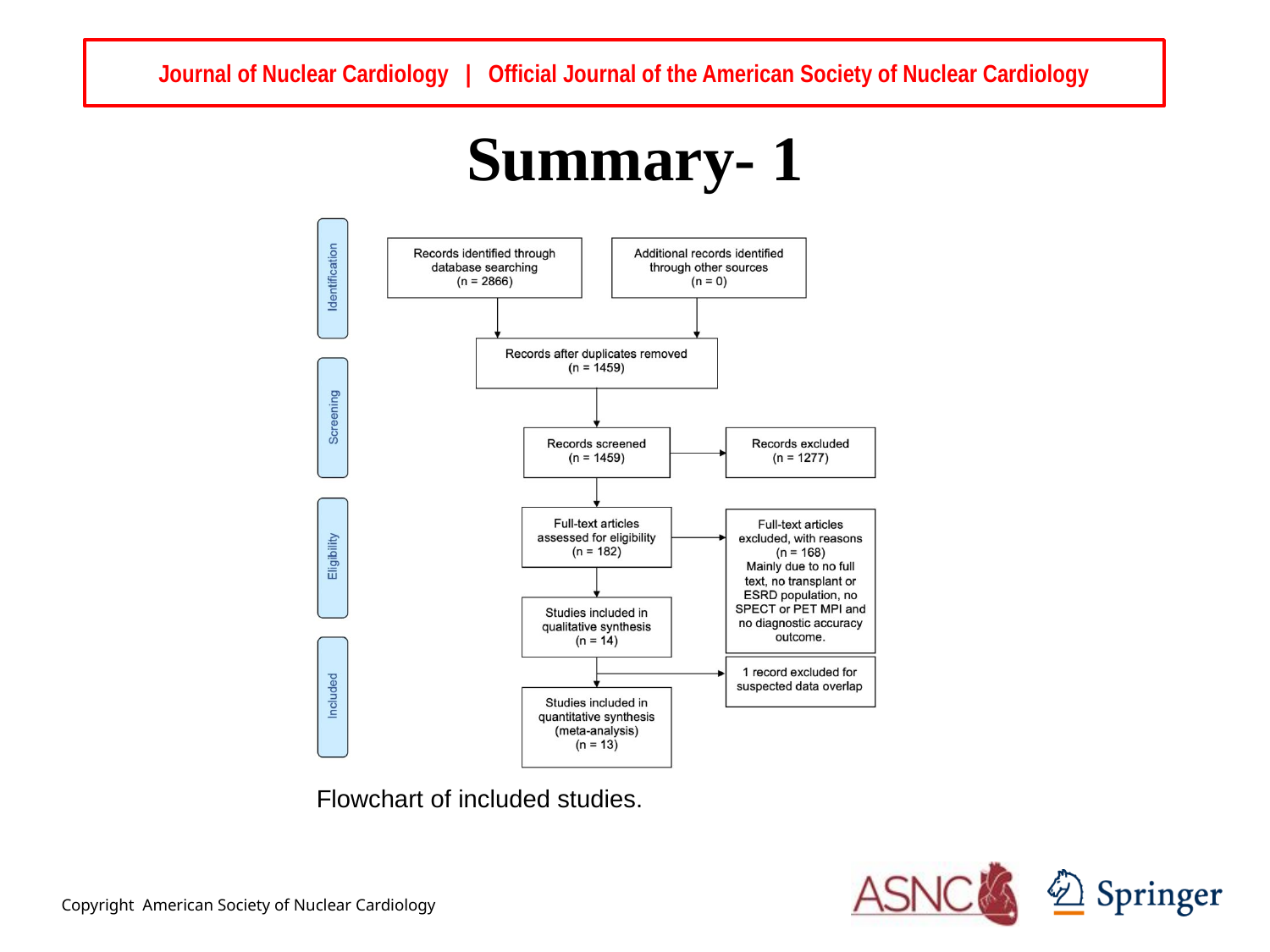

Journal of Nuclear Cardiology | Official Journal of the American Society of Nuclear Cardiology
# Summary- 1
Flowchart of included studies.
Copyright American Society of Nuclear Cardiology

## Slide 4
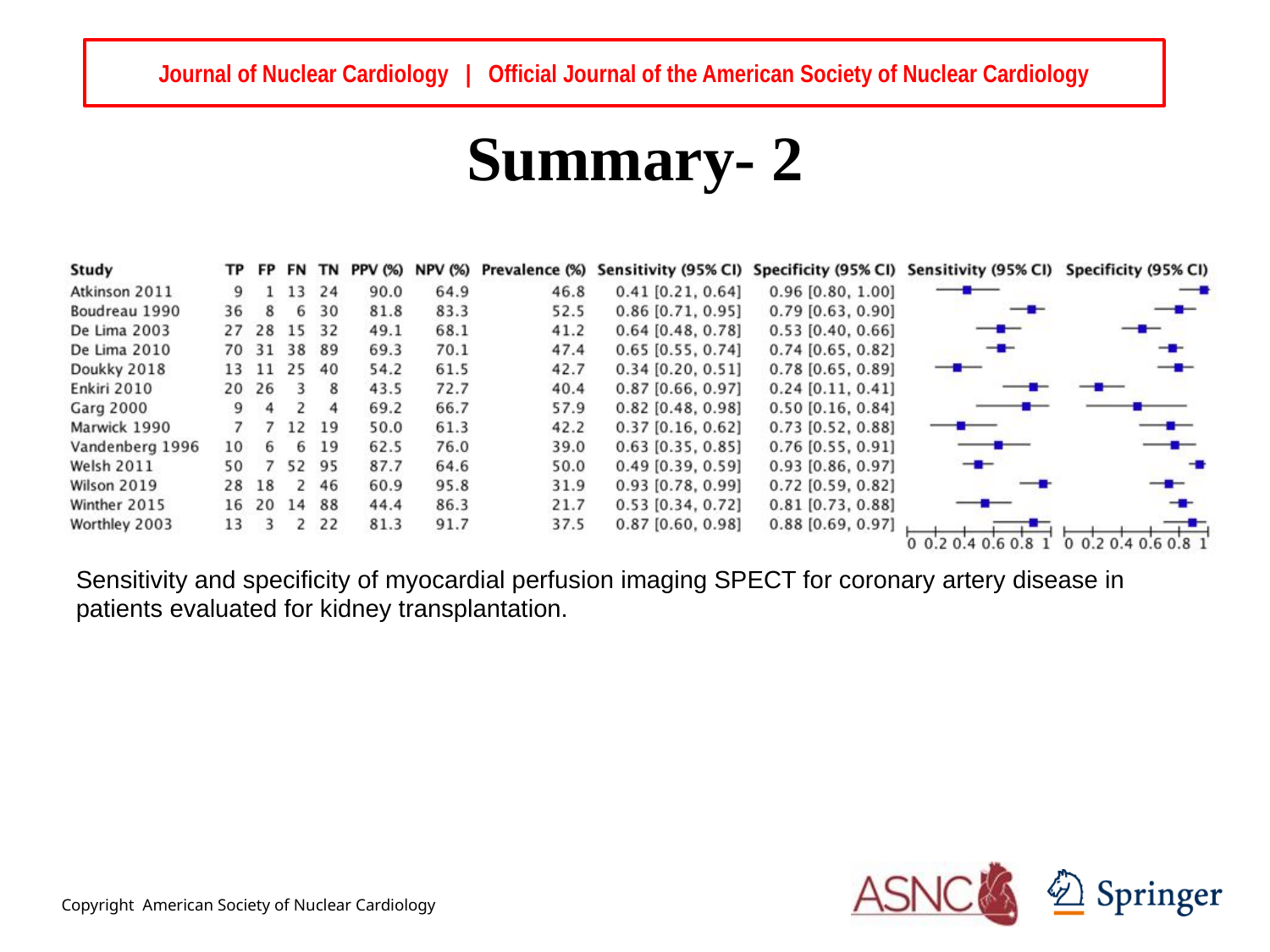

Journal of Nuclear Cardiology | Official Journal of the American Society of Nuclear Cardiology
# Summary- 2
Sensitivity and specificity of myocardial perfusion imaging SPECT for coronary artery disease in patients evaluated for kidney transplantation.
Copyright American Society of Nuclear Cardiology

## Slide 5
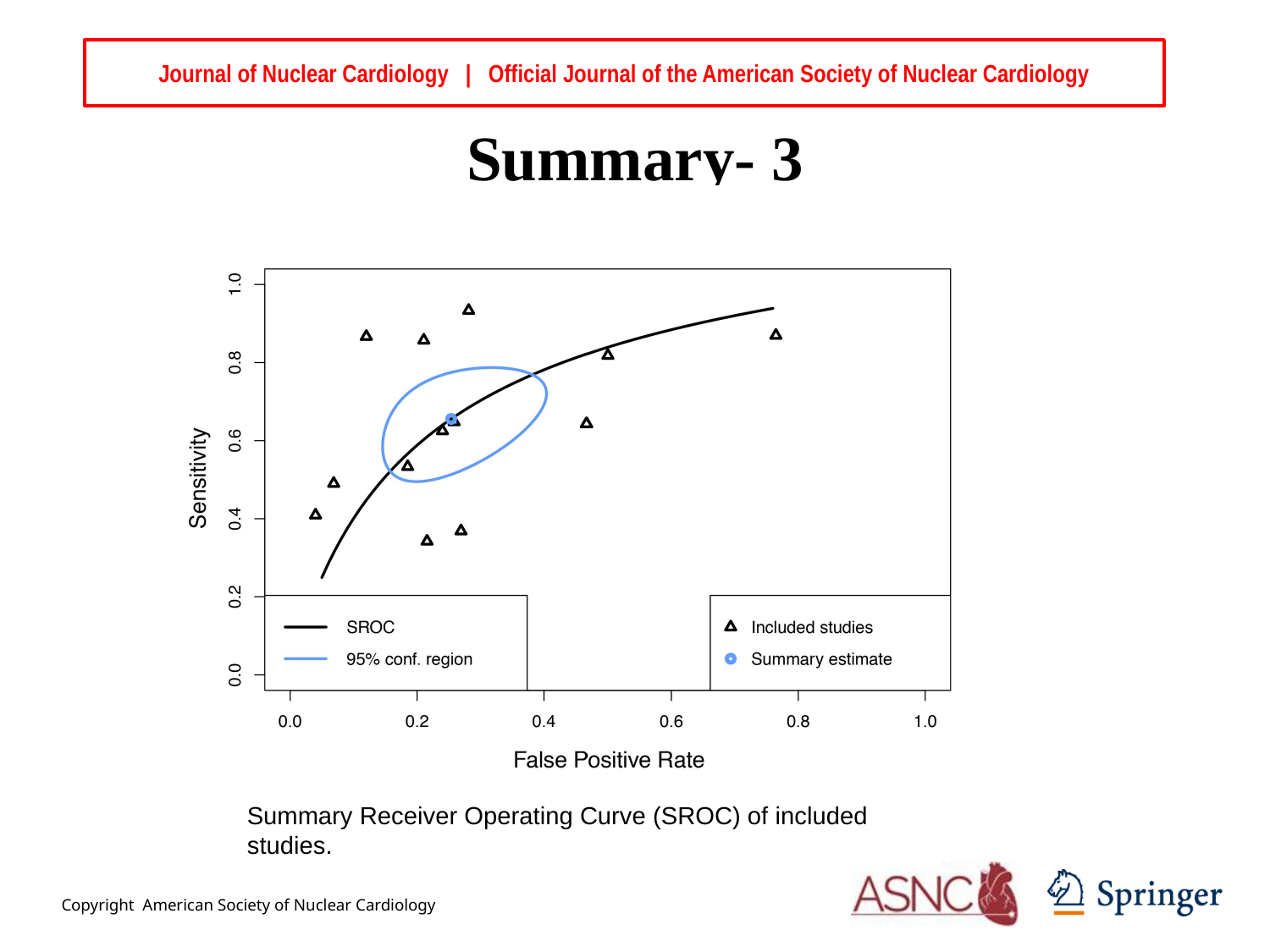

Journal of Nuclear Cardiology | Official Journal of the American Society of Nuclear Cardiology
# Summary- 3
Summary Receiver Operating Curve (SROC) of included studies.
Copyright American Society of Nuclear Cardiology

## Slide 6
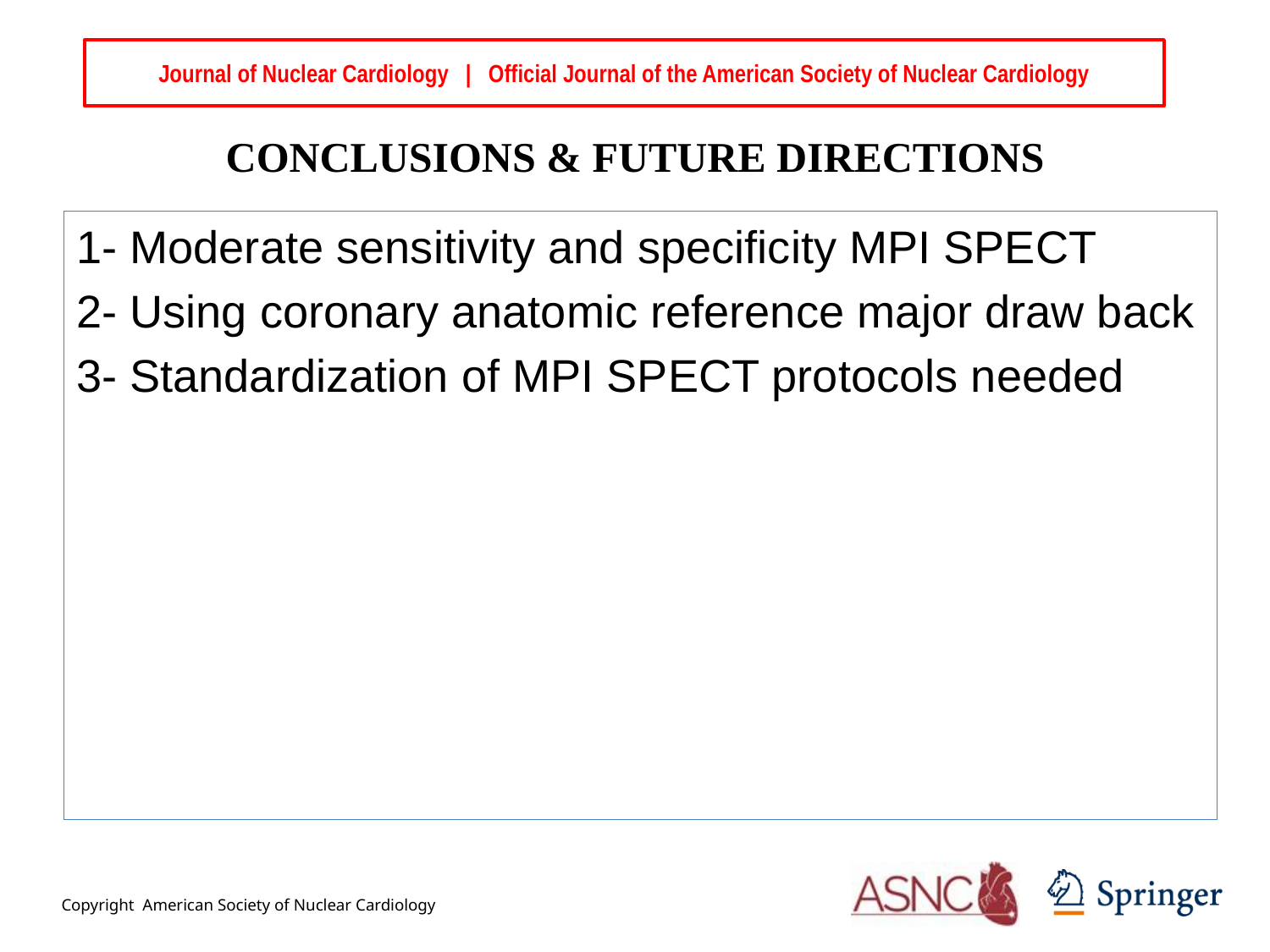

Journal of Nuclear Cardiology | Official Journal of the American Society of Nuclear Cardiology
# CONCLUSIONS & FUTURE DIRECTIONS
1- Moderate sensitivity and specificity MPI SPECT
2- Using coronary anatomic reference major draw back
3- Standardization of MPI SPECT protocols needed
Copyright American Society of Nuclear Cardiology
